# A simplified protocol for the generation of cortical brain organoids

**DOI:** 10.3389/fncel.2023.1114420

**Published:** 2023-04-04

**Authors:** Kristel N. Eigenhuis, Hedda B. Somsen, Mark van der Kroeg, Hilde Smeenk, Anne L. Korporaal, Steven A. Kushner, Femke M. S. de Vrij, Debbie L. C. van den Berg

**Affiliations:** ^1^Department of Cell Biology, Erasmus MC, Rotterdam, Netherlands; ^2^Department of Psychiatry, Erasmus MC, Rotterdam, Netherlands; ^3^Department of Psychiatry, Columbia University Irving Medical Center, New York, NY, United States

**Keywords:** cortical brain organoids, human brain development, self-patterning, dorsal identity, feeder-independent, induced pluripotent stem cells

## Abstract

Human brain organoid technology has the potential to generate unprecedented insight into normal and aberrant brain development. It opens up a developmental time window in which the effects of gene or environmental perturbations can be experimentally tested. However, detection sensitivity and correct interpretation of phenotypes are hampered by notable batch-to-batch variability and low reproducibility of cell and regional identities. Here, we describe a detailed, simplified protocol for the robust and reproducible generation of brain organoids with cortical identity from feeder-independent induced pluripotent stem cells (iPSCs). This self-patterning approach minimizes media supplements and handling steps, resulting in cortical brain organoids that can be maintained over prolonged periods and that contain radial glial and intermediate progenitors, deep and upper layer neurons, and astrocytes.

## 1. Introduction

The human developing brain is unique compared to any other organism due to its increased size, complexity, and expansion of neuronal output (Benito-Kwiecinski et al., 2021). Most of the current understanding of mammalian brain development is derived from studies in rodent models. However, these do not fully recapitulate the dynamic and complex architecture of the human brain nor model the genetic basis of prominent human neurodevelopmental disorders (Velasco et al., 2020). Therefore, in recent years, major advances have been made to generate protocols for *in vitro* differentiation of human pluripotent stem cells (PSCs) into 3D tissue cultures such as cortical organoids, that closely mimic key features of *in vivo* corticogenesis (Lancaster et al., 2013; Camp et al., 2015).

Cortical organoids grow from PSCs that self-aggregate into embryoid bodies and spontaneously acquire neuronal fate, resulting in the formation of polarized neuroepithelial structures (Kadoshima et al., 2013). As described in the initial hallmark protocols (Kadoshima et al., 2013; Lancaster et al., 2013; Lancaster and Knoblich, 2014), supplementing the growth medium of forming organoids with a 3D scaffold (Matrigel) in combination with agitation results in various brain region identities being formed within the organoids in several weeks to months. Defined progenitor zones, containing apical radial glial cells (aRGCs), intermediate progenitor cells (IPCs), and outer radial glial cells (oRGCs), surround ventricle-like lumens and generate deep and upper layer neurons that organize into the cortical plate, the embryonic precursor of the cerebral cortex. In addition to the similar cytoarchitecture, cellular composition, and maturation, cortical organoids have been shown to resemble gene expression profiles of the developing human cortex beyond the second trimester, up to early postnatal stages (Camp et al., 2015; Velasco et al., 2019; Gordon et al., 2021).

Despite their great potential, generating cortical organoids is a delicate and lengthy process, challenged by low reproducibility and sensitivity to perturbations. Therefore, the field is constantly and rapidly developing improved protocols to overcome organoid-to-organoid variability and batch-to-batch variation and to ensure the reproducibility of cell types within single organoids being generated (Velasco et al., 2019). While “self-patterned” generation of cortical organoids as described in initial protocols will spontaneously generate forebrain, midbrain, and hindbrain identities, more recent protocols have focused on guiding or directing toward region-specific identities in organoids, using exogenous patterning factors. Several groups have shown that during embryoid body formation, the addition of WNT (Kadoshima et al., 2013; Mariani et al., 2015; Pasca et al., 2015; Qian et al., 2016; Pollen et al., 2019; Velasco et al., 2019), BMP (Mariani et al., 2015; Pasca et al., 2015; Qian et al., 2016; Xiang et al., 2017), and TGF-β (Kadoshima et al., 2013; Mariani et al., 2015; Xiang et al., 2017; Pollen et al., 2019; Velasco et al., 2019) inhibitors can act as rostralizing signal and promote the generation of (dorsal) forebrain organoids. During neural induction, WNT activators (Lancaster et al., 2017) and Matrigel (Kadoshima et al., 2013; Lancaster et al., 2013) stimulate lateral expansion and budding of the neuroepithelium, respectively. Single-cell RNA-seq on different batches of dorsally patterned forebrain organoids by WNT and TGF-β inhibition has shown that sample-to-sample reproducibility of cell types generated was similar to that between individual endogenous brains (Velasco et al., 2019). However, the addition of small molecules to media compositions can also be a source of variation due to low concentrations added, batch-to-batch variability, and storage conditions.

**Figure 1:**
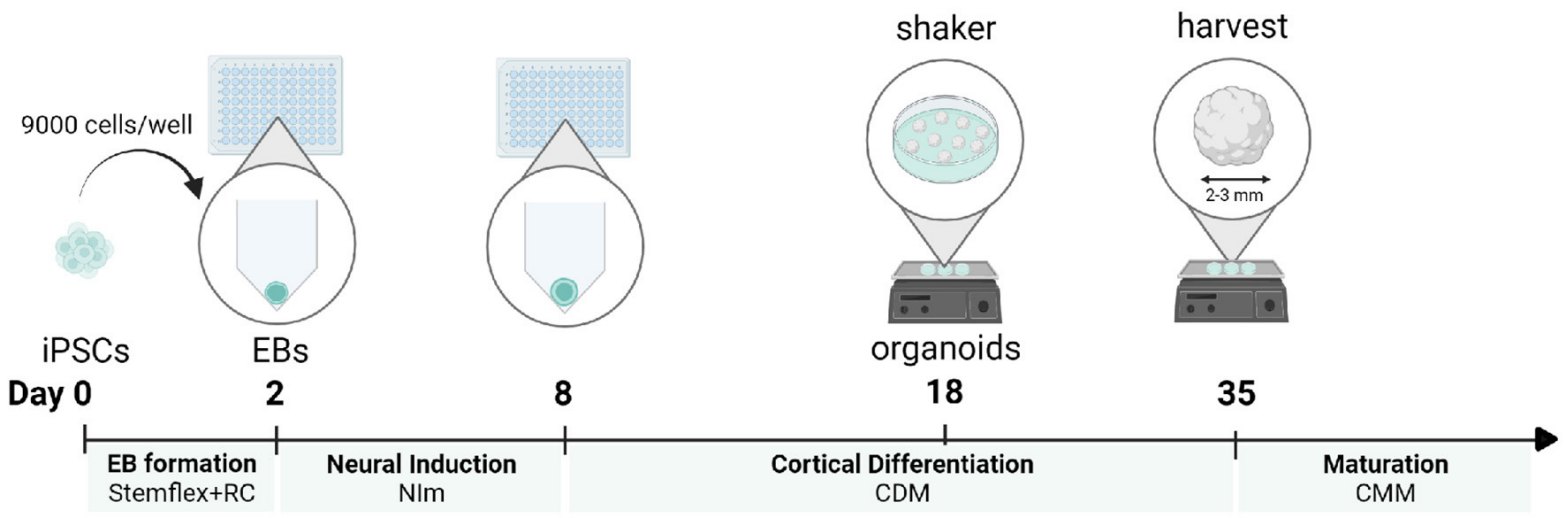
Protocol timeline for a cortical organoid generation. For each developmental stage, the corresponding medium is displayed. iPSCs, induced pluripotent stem cells; EB, embryoid body; NI, neural induction; CDM, cortical differentiation medium; CMM, cortical maturation medium. This figure was created with BioRender.com.

Here, we combine a robust embryoid body-based method for the generation of cortical NPC-derived neural networks (Gunhanlar et al., 2018) with 3D organoid culture to reproducibly generate and mature cortical brain organoids from induced pluripotent stem cells (iPSCs), without the addition of patterning factors (Velasco et al., 2019). In short, 9,000 single-iPSCs derived from feeder-independent cultures are seeded per V-bottom well of a 96-well plate to aggregate into embryoid bodies. Seeding consistent numbers of iPSCs in individual wells in their original culture medium will stimulate the rapid formation of uniform embryoid bodies within 2 days. Subsequently, robust neural induction for 6 days in a simple medium formulation will stimulate the generation of neuroectoderm and neuroepithelial tissues. These tissues acquire a spontaneous preference to develop into brain regions with dorsal forebrain identity. After 18 days of differentiation, formed organoids are transferred into non-adherent dishes and placed on an orbital shaker. Under agitating conditions, the cortical organoids will further grow until they reach a diameter of 2–3 mm after 35 days. At this time point, the organoids can be harvested for immunohistochemical and expression analysis. If preferred, organoids can be further matured by adding Matrigel to the medium to serve as a 3D scaffold (Figure 1). This protocol is optimized to maximize efficiency and minimize the complexity of handling steps, resulting in a practical and reproducible method to generate dorsal forebrain organoids.

## 2. Materials and equipment

### 2.1. Reagents

- WTC-11 human iPSC line (Coriell Repository, GM25256);
- StemFlex™ Medium (ThermoFisher Scientific, #A3349401);
- RevitaCell™ Supplement 100X (ThermoFisher Scientific, #A2644501);
- Geltrex, LDEV-Free, hESC-Qualified, Reduced Growth Factor Basement Membrane Matrix (ThermoFisher Scientific, #A1413301);
- Dulbecco′s Phosphate Buffered Saline (Sigma-Aldrich, #D8537);
- UltraPure™ 0.5M EDTA, pH 8.0 (Invitrogen^TM^, #15575-038);
- StemPro™ Accutase™ Cell Dissociation Reagent (ThermoFisher Scientific, #11599686);
- Advanced DMEM/F-12 (ThermoFisher Scientific, #12634028);
- Penicillin-Streptomycin (Sigma-Aldrich, #P0781);
- N-2 Supplement 100X (ThermoFisher Scientific, 17502048);
- Heparin (Sigma, #H3149);
- Glasgow-MEM, GMEM (ThermoFisher Scientific, #11710035);
- MEM Non-Essential Amin Acids Solution (ThermoFisher Scientific, #11140050);
- DMEM/F-12, HEPES (ThermoFisher Scientific, #11330032);
- GlutaMAX Supplement (ThermoFisher Scientific, #35050061);
- Chemically Defined Lipid Concentrate (ThermoFisher Scientific, #11905031);
- Amphotericin B (ThermoFisher Scientific, #15290018);
- Fetal Bovine Serum, qualified, Brazil (ThermoFisher Scientific, #10270106);
- Matrigel (Corning, #356234);
- Trypan Blue Solution, 0.4% (ThermoFisher Scientific, #15250061);
- Paraformaldehyde (Sigma-Aldrich, #P-6148);
- Sodium hydroxide solution (Sigma, #72068);
- Hydrochloric acid fuming 37% (Sigma, #30721-M);
- TWEEN^®^ 20 (Sigma-Aldrich, #P1379);
- Sucrose (Merck Millipore, #84100);
- Gelatin from porcine skin (Merck Millipore, #04055);
- Trisodium citrate dihydrate (Merck Millipore, #106448);
- Triton^TM^ X-100 (Sigma, #T8787);
- Aqua-Poly/Mount (Polysciences, #18606-20).

### 2.2. Equipment

- PrimeSurface^®^ 3D culture: Ultra-low Attachment Plates: 96-well, V bottom, Clear plates (Sbio Japan, #MS9096VZ);
- Nunc 6-Well Plates, Round (ThermoFisher Scientific, # 140675);
- 5 mL Serological Pipette (VWR, #89130896);
- 10 mL Serological Pipette (VWR, #89130898);
- 15 mL Conical Tubes, Polystyrene (VWR, #352097);
- 50 mL Conical Tubes, Polystyrene (VWR, #352070);
- Hemocytometer (EMS, #68052-14, 68052-15);
- Eppendorf Tubes^®^ 3810X 1.5 ml (Eppendorf, #0030125150);
- Heraeus Megafuge 40R Benchtop Centrifuge (ThermoScientific, #75004519);
- Inverted Phase Contrast Microscope (Olympus CKX31);
- Reagent reservoir, 50 ml (Greiner, #960305);
- 60 mm TC-Treated Cell Culture Dish (Corning, #353002);
- Digital CO_2_-resistant orbital shaker (ThermoScientific, #88881101);
- CO_2_ incubator;
- Multichannel pipet (20–200 μL) (VWR, # 613-2901);
- pH meter (Mettler Toledo, SevenExcellence pH meter S400);
- Glass beaker;
- Stir plate;
- Bijou sample containers (700EA, Sigma-Aldrich, #Z645338);
- Sterile Syringe filters, Pore size 0.2 μm (Cytiva Whatman, #10462200);
- Aluminum PCR heating block;
- Cryomold Bpsy 10 × 10 × 5 mm (Sakura Finetek, #1S-VW-4565-PK);
- SuperFrost Plus^TM^ Adhesion Microscopic Slides (Epredia, #J1800AMNZ);
- ImmunoPen^TM^ (Millipore, #402176);
- Heat-resistant plastic Coplin staining jar (VWR, #470174-652);
- Microscope cover glasses 24 × 60 mm (VWR, #4833 220).

### 2.3. Reagents set-up

#### 2.3.1. Heparin solution

The potency (units/mg) of heparin varies per batch and is reported by the manufacturer on the certificate of analysis. The exact amount of mg present is calculated by dividing the number of units of the batch by the potency in units per mg. The batch is reconstituted in the volume of D-PBS needed to make a 1-mg/ml stock solution (500×). If sterile and filtered through a 0.22-μm filter, this stock solution can be stored at 2–8°C for up to 2 years.

#### 2.3.2. FBS heat inactivation

Heat inactivate serum by placing the bottle in a 56°C water bath for 30 min. Store aliquots at −20°C.

#### 2.3.3. Media preparation

The composition, preparation, and storage conditions of the different media are outlined in Table 1.

**Table 1:** Media preparation.

**Seeding medium (SM)**	**For 10 ml**
StemFlex™ medium	10 ml
1% RevitaCell™ supplement [1X]	100 μl
**Neural induction medium (NIM)**	**For 100 ml**
**Prepare a small aliquot of seeding medium freshly on the day**	
**of seeding. Do not store or re-use**.	
Advanced DMEM/F12 (#12634028)	98 ml
1% Penicillin/streptomycin solution	1 ml
1% N-2 supplement [1X]	1 ml
Heparin [1 mg/ml stock, 2 μg/ml final concentration]	200 μl
**Cortical differentiation medium (CDM)** ^a^	**For 100 ml**
**This medium can be stored and used for 1 week after**	
**preparation, at 4**°**C**.	
DMEM/F-12 (#11330-032)	96 ml
1% GlutaMAX supplement	1 ml
1% Chemically defined lipid concentrate	1 ml
1% N-2 supplement [1X]	1 ml
Amphotericin B [250 μg/ml stock, 0.25μg/ml final]	100 μl
1% Penicillin/streptomycin solution	1 ml
**Cortical maturation medium (CMM)** ^a^	**For 200 ml**
**This medium can be stored and used for 2 weeks after**	
**preparation at 4**°**C**.	
DMEM/F-12 (#11330-032)	169 ml
1% GlutaMAX supplement	2 ml
1% chemically defined lipid concentrate	2 ml
1% N-2 supplement [1X]	2 ml
Amphotericin B [250 μg/ml stock, 0.25μg/ml final]	200 μl
1% penicillin/streptomycin solution	2 ml
10% fetal bovine serum	20 ml
Heparin [1 mg/ml stock, 5 μg/ml final concentration]	1 ml
1% matrigel^*^	2 ml

* Add Matrigel only to cold medium, and only after filtering the medium. If added when the medium is warm, Matrigel will solidify and not properly mix into the solution.

This medium should be filtered before use and can be stored and used for 2 weeks after preparation at 4°C.

a CDM and CMM formulations were originally described as cortical differentiation medium-II (CDM-II) and cortical differentiation medium-III (CDM-III) respectively by: Velasco S. et al., Individual brain organoids reproducibly form cell diversity of the human cerebral cortex, Nature, 2019. Jun; 570(7762): 523–527.

#### 2.3.4. 20% Paraformaldehyde (PFA) stock solution

To prepare 100 ml of 20% PFA stock solution, heat 80 ml of D-PBS in a glass beaker on a stir plate to 55–60°C, in a ventilated hood, Add 20 g of PFA to the heated solution. A cloudy suspension will be the result. Slowly raise the pH by dropwise adding 1M NaOH, until the solution clears. Let the solution cool, filter, and finally, add D-PBS to a volume of 100 ml. Recheck the pH, and adjust with 1 M HCl to pH 6.9. Prepare aliquots and store at −20°C. Dilute 20% PFA in D-PBS to a 4% working solution. Only use 4% PFA on the day of preparation and do not re-freeze or store.

#### 2.3.5. 0.1% PBS-T

To prepare 1 L of 0.1% PBS-T, add 1 ml of Tween^®^ 20 to 1 L of D-PBS and mix thoroughly.

#### 2.3.6. 30% sucrose solution

To prepare 1 L of 30% sucrose solution, weigh 300 g of sucrose and add D-PBS to a final volume of 1 L. Mix using a magnetic stirrer at room temperature until all sucrose has dissolved. Aliquot and store at −20°C.

#### 2.3.7. Gelatin Solution (10% gelatin−7.5% sucrose–PBS)

To prepare 100 ml of gelatin solution, add 10 g of sucrose to 100 ml of D-PBS. To this solution, add 7.5 g of gelatin and mix using a magnetic stirrer at room temperature until both components have completely dissolved. Aliquot and store at −20°C.

#### 2.3.8. Antigen retrieval buffer (10 mM Sodium Citrate, pH 6.0)

To prepare 1 L of Sodium Citrate buffer (10 mM), add 2.94 g of Trisodium citrate dihydrate to 950 ml of dH_2_O. Adjust pH to 6.0 with HCl/NaOH and autoclave. Store at room temperature.

#### 2.3.9. Washing buffer (0.01% Triton X-100–PBS)

To prepare the washing buffer, add Triton X-100 to D-PBS to a final concentration of 0.01%. Store at room temperature.

#### 2.3.10. Blocking buffer/antibody diluent (0.1% Triton X-100–10% FBS-PBS)

To prepare the blocking buffer, add Triton X-100 and FBS to D-PBS to a final concentration of 0.1% and 10%, respectively. Filter using a 0.2-μm syringe filter and store at 4°C for a maximum of 2 days. Prepare freshly on the day of use.

## 3. Methods

### 3.1. Step-by-step procedure: Generation of cortical organoids

#### 3.1.1. iPSC culture

- This protocol describes how to generate cortical organoids from feeder-independent cultured pluripotent stem cells (PSCs). PSCs are cultured in wells of a six-well plate in StemFlex™ Medium, on Geltrex coating ([0.08 mg/ml] final concentration).
- PSCs are cultured in colonies and passaged in clumps every 3–4 days in a 1:4–1:6 ratio by 5′-8′ incubation of 0.5 mM EDTA.
- It is recommended to use PSCs of a passage below 50 and to perform a chromosome count for every 10 passages to confirm a stable karyotype.

#### 3.1.2. Generation of cortical organoids

**Day 0: seeding single iPSCs to generate embryoid bodies—TIMING 1–2 h**

**Figure 2:**
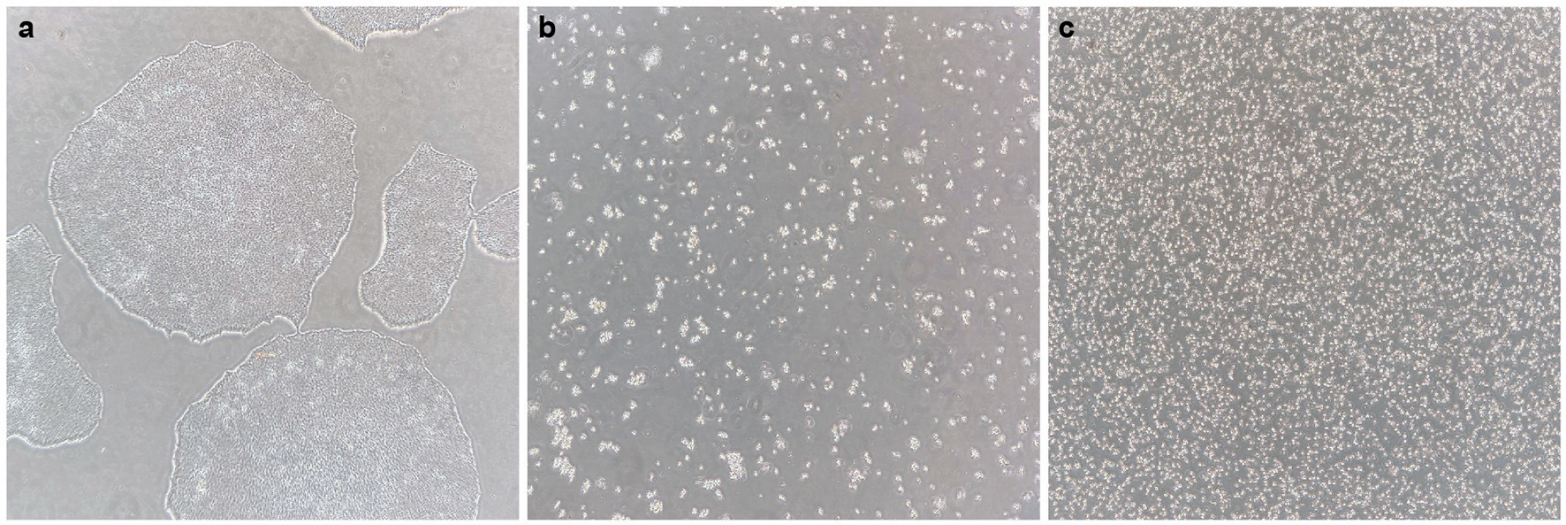
Generating a single-cell iPS suspension. **(a)** Healthy iPS colonies at 70–80% confluency, without signs of differentiation. **(b)** After 0.5 mM EDTA incubation and subsequent incubation with accutase, all cells have dissociated into single cells or small groups of cells, and all cells are floating. **(c)** After gentle trituration of the dissociated cell suspension, a pure single-cell suspension is created.

- It is recommended to seed a maximum of 32 embryoid bodies per batch, to allow for optimal handling conditions.
- 9,000 iPSCs per well are seeded in a 96-well plate to form an embryoid body, in a 200 μL seeding medium.
- To start a batch of 32 EBs, one 80–90% confluent six-well iPSC colony is sufficient. iPSC colonies should not show any sign of differentiation (Figure 2a).

To seed 32 EBs:

1. Prepare 10 ml seeding medium by supplementing 10 ml of StemFlex™ Medium with RevitaCell™ Supplement (1:100). Place in a water bath to warm at 37°C.
2. Wash cells 1× with 1 ml of PBS. To dissociate cells, first, add 1 ml of cold 0.5 mM EDTA per well and incubate for 5′.
3. Aspirate EDTA and add 1 ml of pre-warmed StemPro Accutase per well. Gently disturb the well after 4 min to promote cell dissociation, and incubate for another 4–6 min. All cells should have detached and be floating as single or very small groups of cells (Figure 2b).
4. Collect the cells by pipetting up and down in the Accutase for a maximum of five times with a P1000 pipet. Transfer 1 ml of the cell suspension to a 15-ml falcon tube. Rinse the well with 1 ml seeding medium and add to the collection tube.
5. Quickly count the number of cells/ml by mixing 50 μL of cell suspension with 50 μL of trypan blue. Use a hemocytometer to count cells manually in multiple squares of a counting chamber, and the average will determine the correct cell concentration.

At this stage, it can be observed under the microscope whether a pure single-cell suspension is generated (as shown in Figure 2c). If not, the cell suspension should be gently triturated some more.

6. For 32 EBs, 288.000 (32 × 9,000) cells in 6,400 μL (32 × 200) seeding medium are needed. Taking into account an extra 10%, 316.800 cells in a 7,040-μL seeding medium are needed. Aliquot the required amount of cell suspension into a separate 15-ml falcon tube and centrifuge for 4′ at 200×*g*.
7. Remove the supernatant and resuspend the cell pellet first in 1 ml pre-warmed seeding medium using a P1000, pipetting up and down as little as possible. Add another 6,040 μL of seeding medium to create a cell suspension of the desired concentration.
8. Seed 200 μL cell suspension per well of a 96 v-bottom well plate, using a P1000 pipet. Gently swirl the tube to mix your cell suspension after every eight wells are filled.
9. Place in the incubator at 37°C and 5% CO_2_, on a flat surface, and do not touch or disturb the plate for 48 h.

**Day 2: Refresh embryoid bodies to start germ layer differentiation and neural induction—TIMING 30 min**

**Figure 3:**
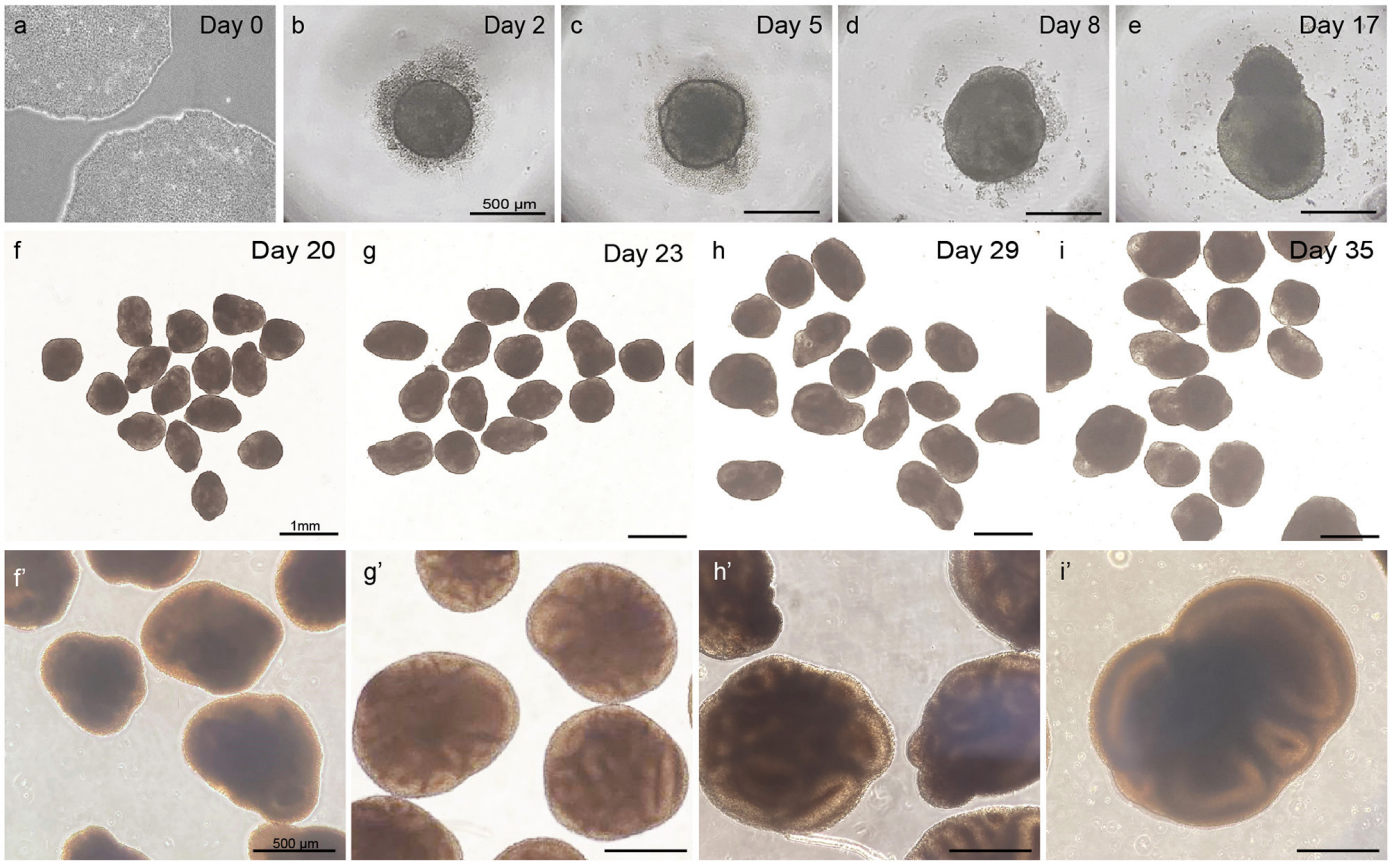
Overview of different steps in embryoid body and cortical organoid development from PSCs. Healthy PSCs **(a)**. The scalebar in panels **(b–e)** represents 500 μm; the scalebar in panels **(f–i)** represents 1 mm; the scalebar in panels **(f′–i′)** represents 500 μm.

Observe whether EBs have formed 48 h after seeding. Ensure to minimize the time of the plate being out of the incubator and minimize disturbance and movement of the plate to retain embryoid bodies in an optimal condition. Round EBs with a clear border and a diameter of 400–500 μm should have formed (Figure 3b). A small number of dead cells likely will surround the EB.

10. Refresh EBs by taking out 180 μL medium/well and adding a fresh pre-warmed 180 μL neural induction medium (NIM)/well. Use a multichannel pipet to remove and add medium, and be cautious not to aspirate EBs into the pipette tips.
11. Return the plate to the incubator and leave it to incubate for another 72 h.

**Day 5: Refresh embryoid bodies with neural induction medium—TIMING 30 min**

When observing at day 5, EBs should have grown to a diameter of 450–550 μm. A clear and defined border should still be visible, and an optically clear and radially organized neurectoderm should have appeared on the outer edge (Figure 3c). Buds of neurectoderm without obvious radial organization can also grow within the EBs, which is acceptable as they will possibly still grow into neuroepithelial structures.

12. Refresh EBs by taking out 180 μL medium/well and adding a fresh pre-warmed 180 μL NIM/well. Use a multichannel pipet to remove and add medium and be cautious not to aspirate EBs into the pipette tips.
13. Return the plate to the incubator and leave it to incubate for 72 h.

**Day 8: Refresh early organoids with cortical differentiation medium—TIMING 30 min**

At day 8, the early organoids should have grown to a diameter of 500–600 μm. The organoids have a denser appearance, and small folds of neuroepithelial structures might be visible on the outer edge. At this stage, the organoid may lose its round shape and adopt a more asymmetrical form. From this day, the medium is switched from NIM to cortical differentiation medium (CDM).

14. Refresh organoids by taking out 180 μL NIM/well and adding a fresh pre-warmed 180 μL CDM per well. Use a multichannel pipet to remove and add medium and be cautious not to aspirate organoids into the pipette tips.
15. Return the plate to the incubator and leave it to incubate for 48 h.

**Day 11–day 18: Refresh organoids with cortical differentiation medium—TIMING 30 min**

16. Refresh organoids three times in 7 days with CDM as described in step 17, leaving a maximum of 48 h in between refreshing steps.

As shown in Figures 3d, e, the neuroepithelium should further grow and form buds that are visible in a translucent and thick edge on the outside of the organoid.

**Day 18: Transfer organoids to 6-cm dishes in cortical differentiation medium and transfer to shaker—TIMING 60 min**

On day 18, the organoids are transferred to non-adherent 6-cm dishes using cut P200 tips. The dishes are placed on an orbital shaker to continue culturing under agitating conditions.

17. First prepare cut P200 tips by cutting off the bottom part of the pipette tip using sterile scissors, creating a tip diameter of ~2 mm. Prewarm the amount of CDM needed. A maximum of 10 organoids is transferred to one ϕ6 cm dish, normally resulting in three ϕ6 cm dishes per batch of organoids seeded. Per dish, 5 ml of CDM is needed.
18. Fill out 5 ml of pre-warmed CDM per ϕ6 cm dish and place it next to the 96-well plate with organoids to be transferred.
19. To pipette up an organoid from its 96-well, place the pipette with the cut P200 tip straight into the well and gently pipette up. It is important not to enter the V-bottom of the well, where the organoid is located. The organoid in the pipette tip will be visible. Let the organoid sink to the bottom of the pipette tip by holding the pipette up straight and finally let it be released in the CDM in the dish, taking along as little adherent medium as possible.
20. Repeat step 19 until 10 organoids are transferred into a dish. Place the dish on an orbital shaker that is placed in an incubator and set it at the right speed. For the CO_2_-resistant orbital shaker used in this protocol (Thermo Scientific™, Cat. 88881102, orbit 1.9 cm), this is 65 rpm. Refer to Box 1 to calculate the correct shaker speed for other shaker types.
21. Leave organoids to incubate under continuous agitating conditions for 48 h.

Box 1 **Calculating shaker speed.**

Shaker speed (in revolutions per minute, RPM) is dependent on the orbit size of the orbital shaker used. For a shaker with an orbit of 1.9 cm, 65 RPM is the desired shaker speed. The correct shaker speed for shakers with a different orbit can be converted using the equation below:

$r2=\sqrt{{\left(r1\right)}^{2}\times \frac{\text{d}1}{\text{d}2}}$

***r1*** **=** shaker speed of first shaker (rpm)

***r2* =** shaker speed of second shaker (rpm)

***d1* =** diameter(throw) of first shaker (cm)

***d2* =** diameter (throw) of second shaker (cm)

For the Thermo Scientific™ (#88881102) orbital shaker used in this protocol:

***r1*** = 65 rpm

***d***_**1**_ = 1.9 cm

**Day 20–35: Refresh organoids with cortical differentiation medium—TIMING 30 min**

Between days 20–35, the CDM medium needs to be refreshed three times per week, leaving a maximum of 48 h in between refreshing steps.

22. To refresh, pause the shaker and transfer dishes to the cell culture hood. Slightly tilt the dishes and pipet off 4.5 ml of medium using a P1000. Avoid disturbance of the organoids in the dish and always leave in 0.5 ml to prevent the organoids from drying out.
23. Slowly add 4.5 ml of fresh and pre-warmed CDM medium.
24. Place dishes back on the shaker and continue incubation under continuous agitation.

As displayed in Figures 3f–i, the organoids should steadily increase in size and density during this phase to a diameter of >800 μm.

**Day 35 and on: Refresh organoids with cortical maturation medium**

From day 35 onward, the medium is changed to cortical maturation medium (CMM). The addition of Matrigel will provide a degree of stiffness to the medium that supports healthy further growth of the organoids.

Be aware to prepare CMM first without Matrigel and filter it using a bottle top filter. After filtering, add Matrigel only if the medium is cold.

25. Refresh organoids three times per week with CMM as described in step 22, leaving a maximum of 48 h in between refreshing steps.

### 3.2. Fixation, embedding, and cryosectioning of cortical organoids

From day 25 onward, the generated organoids can be harvested and processed to observe the developed structures and to verify the presence of dorsal forebrain cell types *via* immunofluorescent staining.

#### 3.2.1. Fixation and equilibration

1. Use a cut-P1000 pipette tip to transfer organoids to a bijou container. Cut a P1000 tip such that the diameter is 3 mm, and ensure that the cutting edge is smooth to prevent organoids from being damaged.
2. Slowly pipette up organoids and collect a maximum of five organoids per container.
3. Aspirate excess medium from the collected organoids and wash by adding 5 ml of D-PBS. Incubate for 5 min and remove D-PBS. Repeat this washing step once more.
4. Add 5 ml of fresh 4% PFA per container and incubate for 4–6 h at 4°C, standing still.
5. Remove PFA from the container and dispose. Wash organoids 2 × 5 min in PBS-T, by adding 5 ml of PBS-T per container.

At this point, organoids can be stored in PBS-T at 2–8°C for up to 1 week. Alternatively, directly continue with sucrose equilibration.

6. To equilibrate organoids, remove PBS-T and discard. Add 5 ml/container of 30% sucrose solution and allow to equilibrate overnight at 2–8°C.

Organoids will float right after the sucrose solution is added. Once the organoids no longer float, equilibration has been successful.

#### 3.2.2. Embedding

Organoids are embedded in gelatin solution in small square embedding molds, and subsequently snap-frozen.

7. Thaw the gelatin solution in a 37°C water bath. Keep the gelatin solution at 37°C continuously to prevent it from polymerizing.
8. Prepare a dry ice/ethanol slurry by spraying 100% ethanol onto dry ice in a styrofoam box multiple times. Once the mixture has stopped boiling, place an aluminum PCR heating block on top of the dry ice and press it down firmly. Wait for 15 min to ensure that the block is ice cold. At this time, let the container with organoids in sucrose solution warm to room temperature.
9. Pipette sucrose solution out of the container containing the organoids and discard. Add 1–2 ml of warm gelatin solution to the organoids and place the container in a small layer of warm water to prevent polymerization.
10. Fill an embedding mold with gelatin solution such that the block is nearly filled. Right after, pipette up organoids with a cut P1000 tip and release them into the embedding mold. Work fast to prevent polymerization of the gelatin. Up to five organoids can be embedded per small embedding mold.
11. The organoids should be positioned at the bottom of the mold, and not be floating. Optionally, use a P200 pipette tip to gently move/reposition the organoids in the mold by hand.
12. If positioned correctly, place the mold on the aluminum block in one movement to snap-freeze. Let it freeze for a minimum of 10 min and do not move the mold during this process.
13. Store snap-frozen samples at −80°C until cryosectioning.

#### 3.2.3. Cryosectioning

14. Remove embedded blocks from the −80°C freezer and allow them to warm up for 30 min in the cryostat chamber that is set at −20°C. Set up the specimen holder temperature at −26°C for gelatin solution-embedded samples.
15. Cut 14–16 μm thick sections and apply them to SuperFrost^®^ Plus adhesion slides. Divide sections over slides in a consecutive sequence to allow for optimal comparison.
16. Let slides air-dry for 15 min before storage at −80°C.

### 3.3. Immunofluorescent characterization of cortical organoid sections

A panel of 12 markers (Table 2) is used to confirm the identity of developed brain regions, as well as cell types that have been generated in cortical organoid sections.

1. Remove slides from the freezer and allow them to dry at room temperature for 15 min.
2. To perform antigen retrieval, place the slides in a plastic coplin staining jar in antigen retrieval buffer (10 mM sodium citrate) and place the jar in a 90°C water bath for 20 min.

**Table 2:** Primary antibodies for cortical organoid characterization.

**Cell type/tissue**	**Antigen**	**Host**	**Supplier**	**Cat. No**.	**Dilution**	**Antigen retrieval**
Radial glia/NSCs	PAX6	Rabbit	BioLegend	901301	1:300	10 mM sodium-citrate, 90°C, 20 min
Forebrain	FOXG1	Rabbit	Abcam	ab18259	1:400	10 mM sodium-citrate, 90°C, 20 min
Intermediate progenitors	TBR2	Rabbit	Abcam	ab23345	1:500	10 mM sodium-citrate, 90°C, 20 min
Neurons	TUJ1	Mouse	BioLegend	801201	1:800	10 mM sodium-citrate, 90°C, 20 min
Deep-layer neurons	CTIP2	Rat	Abcam	ab18465	1:500	10 mM sodium-citrate, 90°C, 20 min
Proliferating cells	Ki-67	Mouse	BD Pharmigen	550609	1:50	10 mM sodium-citrate, 90°C, 20 min
Upper layer neurons	SATB2	Mouse	Abcam	ab51502	1:200	10 mM sodium-citrate, 90°C, 20 min
Astrocytes	GFAP	Rat	Invitrogen	13-0300	1:500	10 mM sodium-citrate, 90°C, 20 min
Cortical neural progenitors and neurons	EMX-1	Rabbit	Atlas antibodies	HPA006421	1:50	10 mM sodium-citrate, 90°C, 20 min
Choroid plexus	TTR	Sheep	BioRad	AHP1837	1:100	10 mM sodium-citrate, 90°C, 20 min
Ventral forebrain progenitors	GSX2	Rabbit	Merck Millipore	ABN162	1:500	10 mM sodium-citrate, 90°C, 20 min
Neuroepithelium and radial glia cells	NESTIN	Mouse	R&D Systems	MAB1259	1:50	10 mM sodium-citrate, 90°C, 20 min

Antigen retrieval is applied to the specific primary antibodies listed in Table 2, but the suitability for antigen retrieval might vary for other antibodies.

3. Let the jar cool to room temperature by opening the lid for 15 min.
4. Pour out the antigen retrieval buffer and replace it with washing buffer, and let it stand for 5 min. Repeat this washing step once more.
5. Place slides on a flat surface (e.g., in a microscope slide box) and pipette 300 μl blocking buffer/slide. Incubate at room temperature for 30 min. Keep the incubation space humidified by placing wet towels.
6. Prepare primary antibody mixes in antibody diluent. Refer to Table 2 for antibody concentrations.
7. Pour off the blocking buffer and optionally mark section areas with an ImmunoPen^TM^. Pipette sufficient primary antibody mix onto slides to cover all slices and incubate overnight in a humidified chamber at 4°C.
8. Prepare secondary antibody mixes in antibody diluent and protect from light. Refer to Table 3 for antibody concentrations.
9. Wash slides 3 × 5 min in a plastic coplin jar in washing buffer.
10. Pipette sufficient secondary antibody mix onto slides to cover all slices and incubate for 1.5 h in a humidified and dark space at room temperature.
11. Pour the secondary antibody mix and wash slides 3 × 5 min in D-PBS in a coplin jar. Keep dark.
12. Quickly dip slides in dH_2_O, add an aqua-poly mount, and cover with a coverslip. Allow to dry overnight in a dark environment at room temperature, and store at 2–8°C before imaging.

**Table 3:** Secondary antibodies.

**Antibody**	**Vendor**	**Cat. No**.	**Dilution**
Alexa Fluor^®^ D α Rt-594	Invitrogen	A-21209	1:1,000
Alexa Fluor^®^ D α Rb-568	Invitrogen	A-11011	1:1,000
Alexa Fluor^®^ Gt α Rb-488	Invitrogen	A-11034	1:1,000
Alexa Fluor^®^ Gt α Ms-488	Invitrogen	A-11029	1:1,000
Alexa Fluor^®^ D α Rb-647	Invitrogen	A-31573	1:1,000
Alexa Fluor^®^ D α Sh-488	Invitrogen	A-11015	1:500
DAPI for nuclei stain	Sigma	D9542	[0.5μg/ml] final

## 4. Anticipated results

This protocol outlines a simplified method to generate cortical organoids from human pluripotent stem cells in which embryoid bodies are formed in the absence of exogenous patterning factors. The resulting organoids will develop brain regions with predominantly dorsal forebrain identity, in a spontaneous matter. This protocol also describes how to examine and characterize generated organoids at various time points and developmental stages.

Per batch of 32 seeded embryoid bodies, typically at least 25 (~80%) correctly developed cortical organoids will have formed after 20 days, which can be further matured for over 100 days. As depicted in Figure 3, at day 5, EBs should exhibit an optically clear and radially organized neurectoderm on the outer edge. In 60–80% of these EBs, the neuroectoderm will further develop and expand after 8 days of culture in a neural induction medium. Subsequent cortical differentiation (days 9–18) should start the generation of neuroepithelial structures inside the EBs, which results in observable growth and increased density of the inner parts. Transferring the EBs to small dishes and the associated change in the medium after 18 days, to further stimulate cortical differentiation, will cause a temporary stagnation of growth for about 2 days. During these 2 days, it is normal to observe slight shrinkage of the now-called organoids. After this critical phase, ~50–80% of the organoids will grow multiple neuroepithelial buds and structures. Neuroepithelial buds can grow in long continuous sheets or round and compact structures, this may vary per organoid and per batch. Budding of neural structures will cause the organoids to steeply increase in size and density, and therefore, it will be difficult to examine the structures by microscope from this phase on. Common pitfalls that might occur during the differentiation of PSCs to organoids, possible explanations, and suggested solutions are described in the troubleshooting table below (Table 4).

**Table 4:** Troubleshooting.

**Days**	**Problem**	**Possible explanation**	**Suggested solution**
0–2	No EB formation	Too low cell viability/unhealthy PSCs	Check PSC pluripotency by microscopic assessment and by staining for OCT3/4 and NANOG markers.
			Perform a karyotype analysis/chromosome count to assess chromosome stability.
	Too small/too big EBs	Inaccurate cell counting	Count cells manually using a hemocytometer
	Much cell death	Too harsh pipetting causing dead cells	Work as fast as possible in generating a single cell suspension, and limit pipetting of cells with a P1000.
2–8	No formation of neuroectoderm	Cells are not pluripotent or EBs are too immature for neural induction	Check PSC pluripotency by microscopic assessment and by staining for OCT3/4 and NANOG markers.
			Healthy and mature EBs should have a uniform round morphology and exhibit defined edges. Too many dead cells surrounding EBs indicate an unhealthy state.
8–18	No formation of neuroepithelial buds	Failed neuroepithelium development	Do not continue culturing these organoids
18–35	Growth arrest of organoids	Change of medium and transfer to agitating conditions	Wait for organoids to recover and grow again after 2–3 days
	Organoids aggregate and collapse	Shaker speed is too low or too many organoids per 6 cm dish	Calculate the correct shaker speed using Box 1 and try different speeds.
			Lower the number of organoids per 6-cm dish
	Organoids break apart/shatter	Shaker speed is too high	Calculate the correct shaker speed using Box 1 and try different speeds.

**Figure 4:**
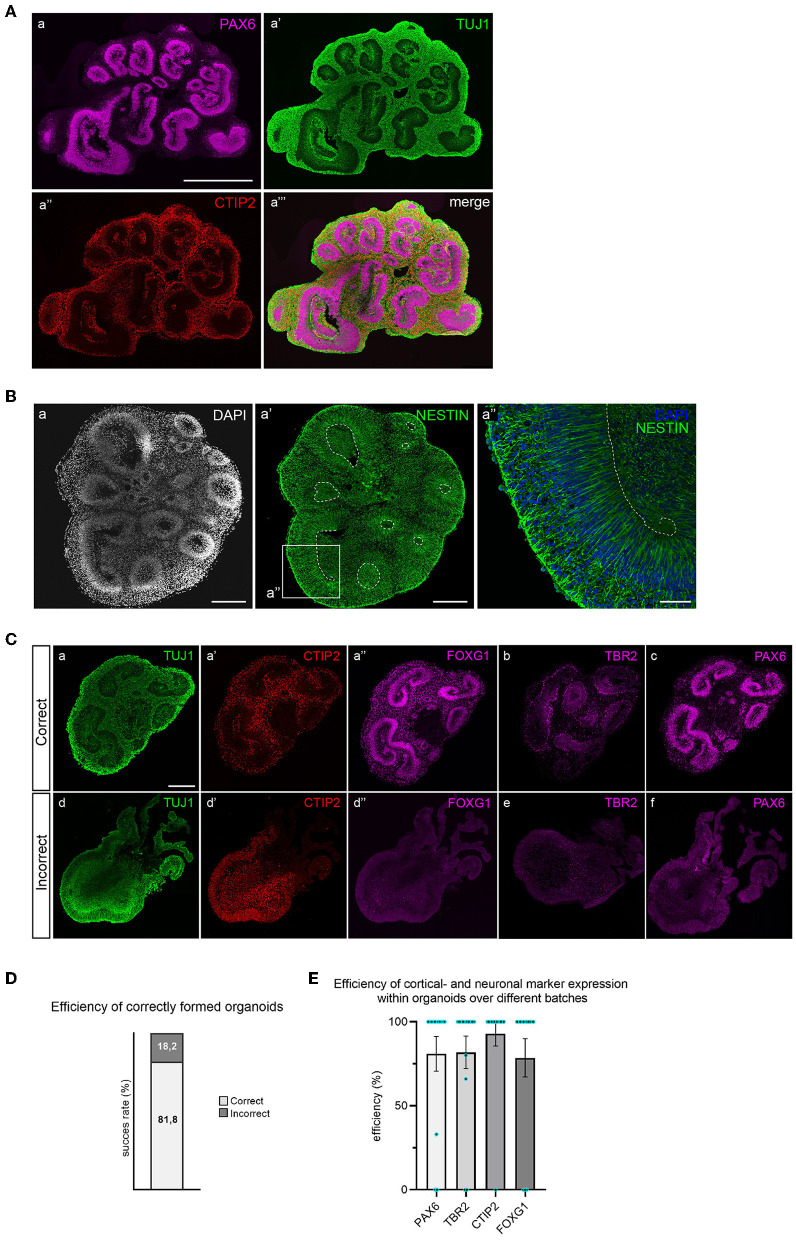
Immunofluorescent staining for brain regions and neuronal cell types in 35-day old cortical organoids. **(A)** Multiple PAX6^+^ progenitor zones are distributed throughout the organoid in the shape of large folds or small rosettes, and surrounding ventricle-like cavities. Whereas progenitor zones are negative for TUJ1 (class-III tubulin), early neuronal differentiation outside of progenitor zones is marked by abundant TUJ1 expression. Early-born deep-layer neurons (CTIP2) reside outside of progenitor zones. Scalebar: 1 mm for all panels. **(B)** Apical radial glial cells (NESTIN^+^) occupy progenitor zones and extend from the apical to the basal surface (panel a″). Scalebar a, a′: 250 μm, a″: 50 μm. **(C)** Correctly formed organoids show structured progenitor zones (panel a), presence of deep-layer neurons (CTIP2, panel a′), and expression of dorsal forebrain markers FOXG1, TBR2, and PAX6 (panels a″, b, and c). Incorrect organoids lack organized progenitor zones (panel d), contain dispersed deep-layer neurons (panel d′), and show absence of dorsal forebrain progenitor markers (panels d″, e, and f). Scalebar: 250 μm for all panels. **(D)** Batch efficiency of brain organoid differentiation. **(E)** Efficiency of cortical identity acquisition.

After 35 days of differentiation, cortical organoids should have developed multiple PAX6^+^ progenitor zones in the shape of rosettes or larger continuous folds (Figure 4A). Progenitor zones form around ventricle-like cavities and are characterized by the absence of neurons (TUJ1 negativity) and the presence of NESTIN-positive aRGCs (Figure 4B). Neuronal differentiation can be recognized by the early neuronal marker TUJ1 and by the presence of early-born deep-layer neurons (CTIP2^+^) that reside outside of the progenitor zones (Figure 4A).

Since different types of brain regions may have formed, a combination of TBR2, FOXG1, and PAX6 staining is used to confirm dorsal forebrain identity (Figure 4C). Correctly formed organoids exhibit organized progenitor zones (TUJ1 negative) that contain FOXG1- and PAX6-expressing radial glial progenitor cells, surrounded by intermediate progenitor cells (TBR2^+^). Deep-layer neurons will locate outside previously formed, radially organised progenitor zones. The efficiency of correctly formed organoids is ~80% (Figure 4D). Incorrectly formed organoids lack the proper structure of progenitor zones, visible by increased TUJ1 signal and loss of radial organization (Figure 4C). These organoids typically do not express dorsal progenitor markers FOXG1, TBR2, or PAX6. CTIP2-positive neurons are often present in incorrectly formed organoids, but lack organized localization (Figure 4C). The efficiency of cortical- and neuronal marker expression within organoids over different batches is displayed in Figure 4E. An in-depth overview of the batch success rate and expression of these different markers across individual batches is provided in Supplementary Table 1.

**Figure 5:**
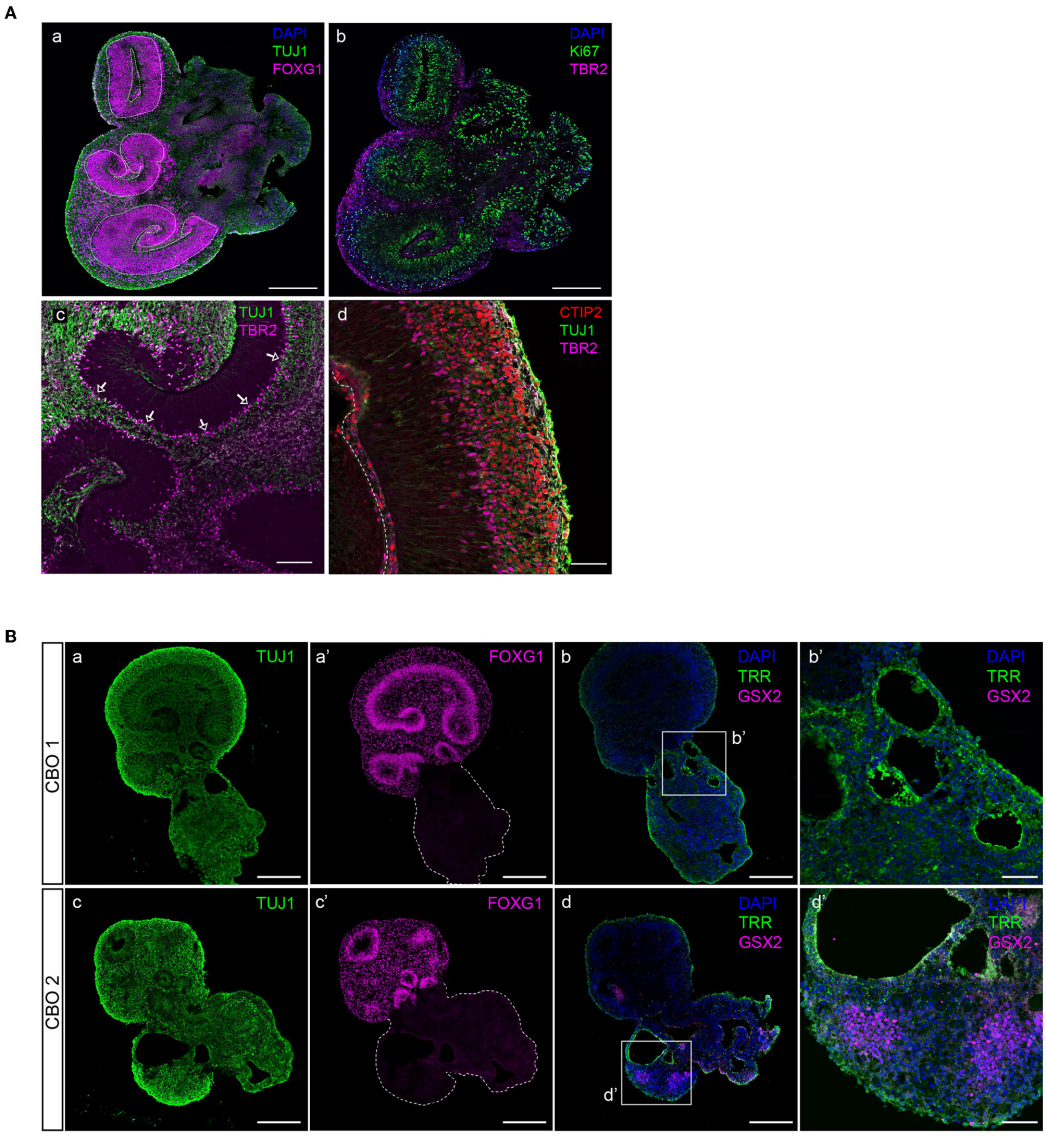
Characterization of dorsal forebrain identity in cortical organoid brain regions. **(A)** A 35-day-old organoid showing multiple radial glial progenitor regions (indicated by dashed lines) marked by FOXG1-positive neural progenitor cells, and early neuronal differentiation (TUJ1) (panel a), proliferative progenitors (Ki-67, panel b), and IPCs (TBR2, panel b). Intermediate progenitor cells (TBR2) are generated in the proliferative zone and migrate to align at the basal side (indicated by arrows), where they generate neurons (TUJ1) (panel c). Magnification of a typically organized proliferative zone (TUJ1 negative) located next to a ventricle-like cavity with intermediate progenitors (TBR2) that give rise to deep-layer neurons (CTIP2) that form a layer right above (panel d). Scalebar a, b: 300 μm, c: 100 μm, d: 50 μm. **(B)** Non-dorsal forebrain identity regions can be recognized by aberrant TUJ1 morphology, lacking progenitor zones (panels a, c), or FOXG1 expression (panels a′ and c′). The presence of ventral forebrain progenitors (GSX2) and choroid plexus epithelia (TRR) surrounding ventricle-like cavities can be identified (panels b, b′, d, and d′). CBO, cortical brain organoid. Scalebar a, a′, b, c, c′, and d: 250 μm, b′ and d′: 50 μm.

Dorsal forebrain progenitor cells are marked by nuclear FOXG1 expression (Figure 5A). Forebrain progenitor zones are highly proliferative, as shown by positivity for proliferation marker Ki-67 (Figure 5A). Consistent with Ki-67 expression reaching a maximum in mitosis, its levels are highest near the apical surface where mitosis takes place following interkinetic nuclear migration (Bruno and Darzynkiewicz, 1992; Kulikova et al., 2011).

In addition to FOXG1 and PAX6 expression in aRGCs, the presence of TBR2-expressing IPCs confirms dorsal forebrain identity (Figure 5A). IPCs align at the outer edge of progenitor zones and are the main source of the early deep-layer neurons (CTIP2) that will settle outside of the progenitor zones (Figure 5A). However, infrequent brain regions with non-dorsal identity might develop in organoids. These regions are morphologically different from dorsal forebrain regions, lack clear progenitor zones, and do not express dorsal forebrain marker FOXG1 (Figure 5B). Immunofluorescent characterization shows that such regions contain ventricle-like cavities surrounded by choroid plexus epithelia (TRR^+^), as well as GSX2-expressing ventral forebrain progenitor cells (Figure 5B), which are both cell types that derive from the rostral neural tube during early embryonic development (Liddelow, 2015; Leung et al., 2022).

**Figure 6:**
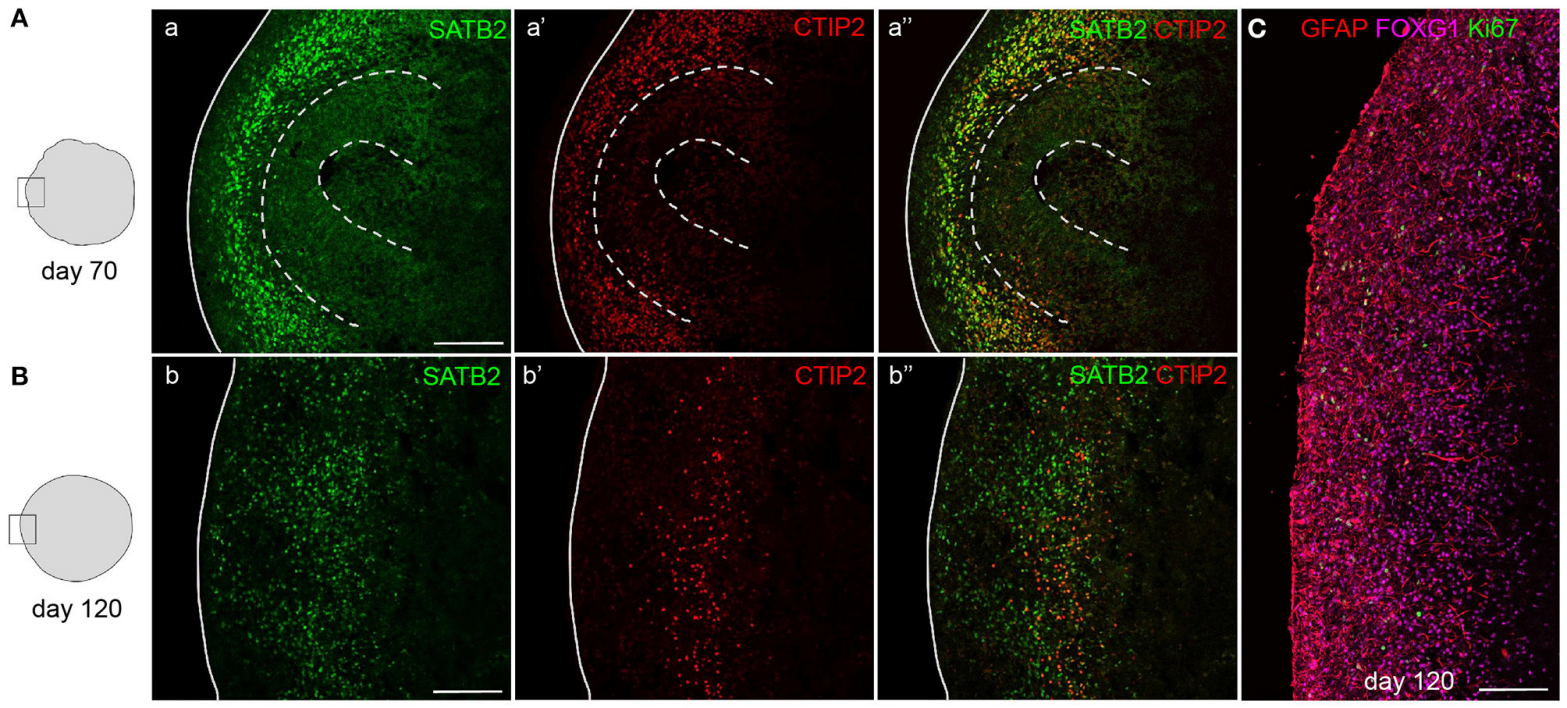
Mature cortical organoids. Staining for upper-layer neurons (SATB2) and deep-layer neurons (CTIP2) in 70- and 120-day-old mature cortical organoids. After 70 days, progenitor zones are still present (indicated by dashed lines) and both types of neurons reside outside of these zones in a disorganized manner **(A)**. In 120-day-old organoids, progenitor zones are absent and deep-layer neurons have compacted into a more organized layer, positioned below a layer of abundantly present upper-layer neurons **(B)**. Staining for astrocytes (GFAP), neurons (FOXG1), and proliferating cells (Ki-67) in a 120-day-old cortical organoid cortex-like region **(C)**. Scalebar: 150 μm in all images.

Whereas, newborn neurons at early stages can still be dispersed, and more compact and organized bands of neurons will be visible in mature organoids (Figure 6). After 70 days of differentiation, mature organoids have expanded their neuronal population with later-born superficial-layer identity neurons (SATB2^+^) having been generated in addition to deep-layer neurons (CTIP2^+^) (Figure 6A). At this stage, both types of neurons settle outside the still-present ventricular zone structures in an intermingled manner. If allowed to further mature up to 120 days *in vitro*, a more structured organization of neurons can be observed, with early-born neurons forming a compact deep layer and later-born neurons being localized outside of this layer (Figure 6B). Progenitor zones gradually thin and eventually disappear, as indicated by the near-absence of Ki-67^+^ cells (Figure 6C). The maturity of 120-day-old organoids is confirmed by the presence of glial cell populations such as astrocytes. As shown in Figure 6C, GFAP^+^ astrocytes are widely distributed throughout the cortex-like organoid tissue, while post-mitotic neurons still express FOXG1 to promote cell survival.

## 5. Discussion

We have described a simple protocol for the generation of cortical organoids from human pluripotent stem cells. The particular focus of this protocol has been to minimize the complexity of handling steps and media formulations while describing a robust and reproducible method. Organoids generated with this protocol were shown to contain brain regions with a cortical identity by the presence of intermediate progenitor cells and NPCs expressing forebrain marker FOXG1. During a differentiation period of 35 days, three different medium formulations are used, and only one switch in culture ware is required. Furthermore, no exogenous patterning factors are added to the culture medium, contributing to the straightforwardness of this protocol.

Various existing protocols have described the differentiation of pluripotent stem cells into embryoid bodies, and subsequent neural induction steps to generate neural stem cells, neural rosettes, or cortical organoids (Kadoshima et al., 2013; Lancaster and Knoblich, 2014; Renner et al., 2017; Sloan et al., 2018; Velasco et al., 2019). *In vitro* modeling of neurogenesis heavily relies on the intrinsic bias of embryonic stem cells toward the neural lineage and their spontaneous preference to differentiate into neuroectoderm and forebrain identity (Gaspard et al., 2008). Where initial brain organoid protocols described self-patterning methods, more recent protocols have focused on directing toward region-specific identities in organoids, using exogenous patterning factors. Mainly the addition of certain inhibitors (TFGβ, BMP, and WNT) is suggested to increase neural induction efficiency and block non-neural differentiation (Chiaradia and Lancaster, 2020). However, we show that robust neural induction of embryoid bodies is sufficient to stimulate the formation of neuroectoderm that will grow into neuroepithelial structures. We describe the formation and neural induction of embryoid bodies *via* a procedure that is very similar to that established by Gunhanlar et al. (2018), where neural induction of embryoid bodies in a simple medium without patterning factors results in NPCs with dorsal forebrain identity. These NPCs could be further differentiated into electrophysiologically mature cortical neuronal networks. A substantial improvement compared to this and other protocols is that we aggregate pluripotent stem cells in a PSC culture medium. This ensures the robust formation of embryoid bodies and minimizes extrinsic disturbance during the fragile process of neural induction. This protocol is in concept comparable to early hallmark protocols that have described the self-patterned generation of cortical organoids (Lancaster et al., 2013; Lancaster and Knoblich, 2014), but is simplified in several ways. In the initial protocols, embryoid body formation, neural induction, and cortical differentiation require four changes of culture ware and four different medium formulations within 15 days of differentiation. Furthermore, neuroepithelial bud expansion requires transferring of early organoids into Matrigel droplets, a vulnerable and time-consuming step. The protocol that we describe uses three different medium formulations and involves only one change in culture ware. In line with published brain organoid differentiation methods (Kadoshima et al., 2013; Velasco et al., 2019), the protocol that we describe incorporates a low concentration of Matrigel in the medium to provide a degree of stiffness that will support the 3D expansion of neuroepithelial structures, without having to incorporate an embedding step.

Since this protocol is focused on the practicality of differentiation conditions and handling, it creates certain limitations for specific downstream applications. To our opinion, generating relatively small amounts of organoids per batch contributes to accurate execution and thereby reproducibility of the protocol. By limiting the time that developing organoids are taken out of their incubator (e.g., to refresh media or take microscopic images), the efficiency at which well-developed organoids are being formed is increased. However, the small number of organoids being generated per batch can be limiting if certain effects need to be studied in larger groups of samples at the same time. Formation of non-dorsal forebrain identity brain regions may occur throughout organoid batches more variably compared to directed protocols, which could be a disadvantage for gene expression analysis such as (single-cell) RNA sequencing.

## Data availability statement

The original contributions presented in the study are included in the article/Supplementary material, further inquiries can be directed to the corresponding author.

## Author contributions

KE: conceptualization, methodology, investigation, formal analysis, and writing—original draft. HBS and AK: investigation. MK and HS: methodology. SK: supervision and funding acquisition. FV: writing—review and editing and supervision. DB: conceptualization, methodology, writing—review and editing, supervision, and funding acquisition. All authors contributed to the article and approved the submitted version.

## Supplementary material

Supplementary Figure 1 Cortical brain identity is marked by EMX-1 expression in organoids. Immunofluorescent staining of a 35-day organoid for dorsal forebrain progenitor marker EMX-1. Top: entire organoid, bottom: magnification view of panel b.

Supplementary Table 1 Efficiency of correctly formed organoids and expression of cortical and neuronal markers within and between batches.
