# Supplementary figures and images for: A simplified protocol for the generation of cortical brain organoids
Source: Front Cell Neurosci. 2023 Apr 4;17:1114420. doi: 10.3389/fncel.2023.1114420 (PMC10110973; doi:10.3389/fncel.2023.1114420)

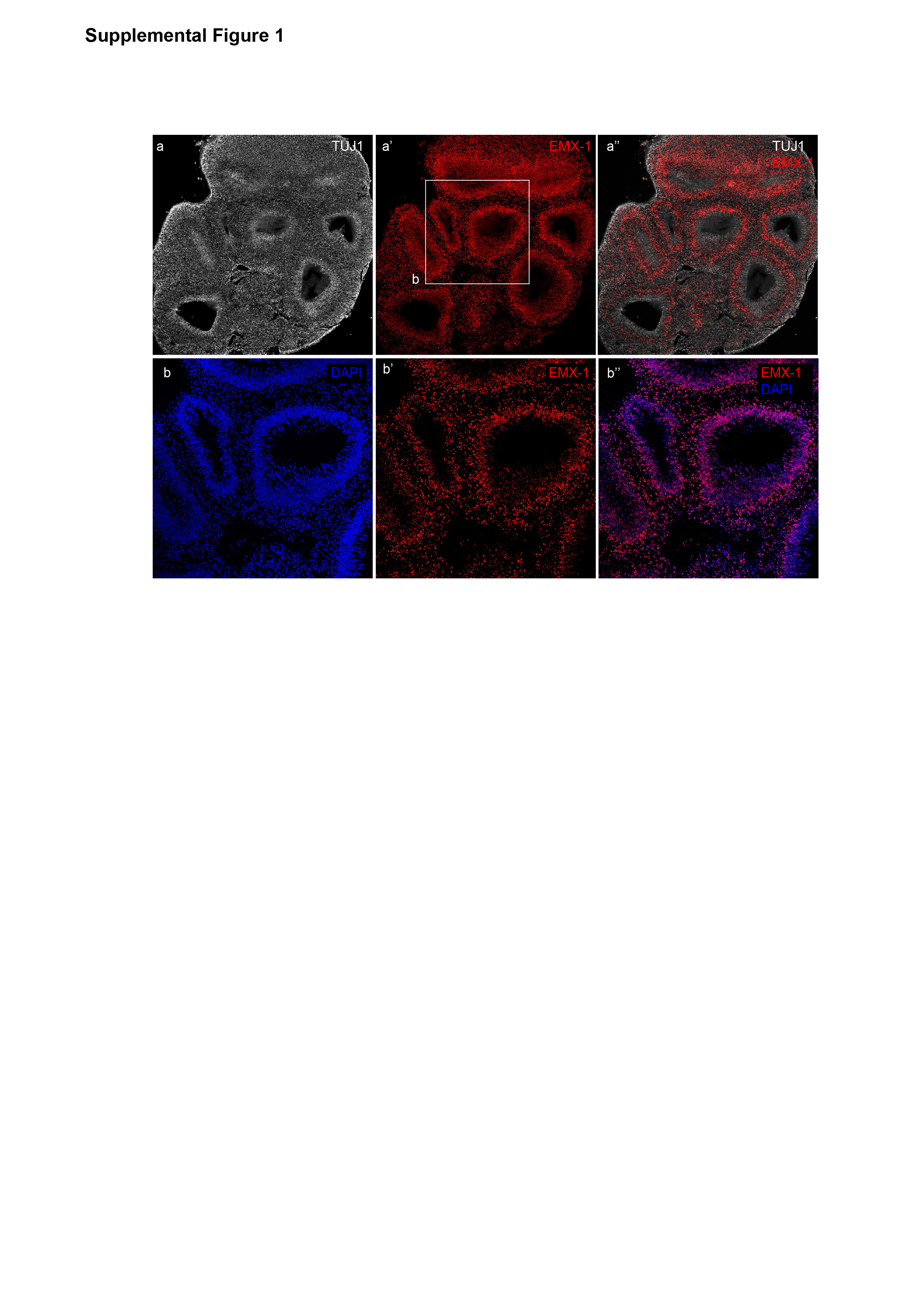

Supplement: Supplementary Figure 1 — Cortical brain identity is marked by EMX-1 expression in organoids. Immunofluorescent staining of a 35-day organoid for dorsal forebrain progenitor marker EMX-1. Top: entire organoid, bottom: magnification view of panel b. [file Image_1.JPEG]
