# Supplementary material for: A simplified protocol for the generation of cortical brain organoids
Source: Front Cell Neurosci. 2023 Apr 4;17:1114420. doi: 10.3389/fncel.2023.1114420 (PMC10110973; doi:10.3389/fncel.2023.1114420)
Supplement: Supplementary Table 1 — Efficiency of correctly formed organoids and expression of cortical and neuronal markers within and between batches. [file Table_1.docx]

| **Efficiency of correctly formed organoids** | | | | |  |  | **Expression of cortical and neuronal markers by IF** | | | | | | | |
| --- | --- | --- | --- | --- | --- | --- | --- | --- | --- | --- | --- | --- | --- | --- |
| **Batch n°** | **Time point of analysis (days *in vitro*)** | **Number of organoids analyzed** | **Correct structure (TUJ1)** | **Efficiency** |  |  | **FOXG1** | **Efficiency** | **PAX6** | **Efficiency** | **TBR2** | **Efficiency** | **CTIP2** | **Efficiency** |
| 1 | 36 | 5 | 5/5 | 100% |  |  | 5/5 | 100% | 5/5 | 100% | 5/5 | 100% | 5/5 | 100% |
| 2 | 36 | 3 | 0/3 | 0% |  |  | 0/3 | 0% | 0/3 | 0% | 0/3 | 0% | 0/3 | 0% |
| 3 | 36 | 3 | 3/3 | 100% |  |  | 3/3 | 100% | 3/3 | 100% | 3/3 | 100% | 3/3 | 100% |
| 4 | 36 | 3 | 3/3 | 100% |  |  | 3/3 | 100% | 3/3 | 100% | 3/3 | 100% | 3/3 | 100% |
| 5 | 36 | 1 | 0/1 | 0% |  |  | 0/1 | 0% | 0/1 | 0% | 0/1 | 0% | 1/1 | 100% |
| 6 | 41 | 3 | 3/3 | 100% |  |  | 3/3 | 100% | 3/3 | 100% | 3/3 | 100% | 3/3 | 100% |
| 7 | 35 | 4 | 4/4 | 100% |  |  | 4/4 | 100% | 4/4 | 100% | 4/4 | 100% | 4/4 | 100% |
| 8 | 34 | 3 | 0/3 | 0% |  |  | 0/3 | 0% | 1/3 | 33,3% | 2/3 | 66,6% | 3/3 | 100% |
| 9 | 32 | 2 | 2/2 | 100% |  |  | 2/2 | 100% | 2/2 | 100% | 2/2 | 100% | 2/2 | 100% |
| 10 | 45 | 3 | 3/3 | 100% |  |  | 3/3 | 100% | 3/3 | 100% | 3/3 | 100% | 3/3 | 100% |
| 11 | 36 | 4 | 4/4 | 100% |  |  | 4/4 | 100% | 4/4 | 100% | 4/4 | 100% | 4/4 | 100% |
| 12 | 35 | 5 | 4/5 | 80% |  |  | 5/5 | 100% | 5/5 | 100% | 4/5 | 80% | 5/5 | 100% |
| 13 | 42 | 2 | 2/2 | 100% |  |  | 2/2 | 100% | 2/2 | 100% | 2/2 | 100% | 2/2 | 100% |
| 14 | 42 | 3 | 3/3 | 100% |  |  | 3/3 | 100% | 3/3 | 100% | 3/3 | 100% | 3/3 | 100% |
|  |  |  |  |  |  | **Overall**  **efficiency** | 37/44 | 84% | 38/44 | 86% | 38/44 | 86% | 41/44 | 93% |
| **Total** |  | 44 |  |  |  |  |  |  |  |  |  |  |  |  |
| **Batch efficiency** |  |  | 11/14 | 79% |  |  |  |  |  |  |  |  |  |  |
| **Overall organoid efficiency** |  |  | 36/44 | 82% |  |  |  |  |  |  |  |  |  |  |
